# A Copy of a Letter from His Excellency the Lord Lieutenant of Ireland to John Julius Angerstein, Esq. Chairman of the Committee of the Royal Jennerian Society for the Extermination of the Small Pox

**Published:** 1803-06-01

**Authors:** 

**Affiliations:** Dublin Castle


*A Copy of a Letter from his Excellency the Lord Lieutenant of Ireland to* JOHN JULIUS ANGERSTEIN, *Esq. Chairman of the Committee of the Royal Jennerian Society for the* EXTERMINATION *of the* SMALL POX.

SIR,

BEFORE I received the letter of the 29th ult. which you signed as Chairman of the Committee of the Jennerian Society for the Extermination of the Small-pox, I had endeavoured to obtain some information upon the measures which had been adopted, for extending the benefits of your Institution to Ireland; and particularly to ascertain, in what mode the assistance of Government could be best applied towards promoting an object, which is upon every account so interesting to the public.

I understand from the Medical Gentlemen of Dublin, whom I have consulted on the subject, that it is very important to ascertain the best mode of obtaining and preserving the genuine Vaccine matter: I have therefore directed a letter to be written upon the subject to the President of the College of Physicians, of which a copy is enclosed; and shall be glad to receive any information and assistance which you may think it useful to communicate.

I shall be extremely happy to accept the office of Vice-President of the Jennerian Society; and beg leave to return you my acknowledgements, for the manner in which you have been so good as to propose it.

I remain, &c.

*Dublin Castle, Feb.* 18. 1803. HARDWICKE

May it please your Excellency,

THE Medical Council of the Royal Jennerian Society for the Extermination of the Small-pox, have directed us to answer the letter, which your Excellency did Mr. Angerstein the honour to address to him as Chairman of the Committee of the Society, relative to the best mode of obtaining and preserving the genuine Vaccine matter.

This office we cheerfully undertake; sensible of the advantage which must accrue to the public, when Vaccine Inoculation is practised under such auspices, and when the assistance of Government is applied to the promotion of that object.

When fluid matter can be procured, it is always to be prefered; especially when the Small-pox prevails in any district where Vaccine Inoculation is to be introduced. Some persons who are to be inoculated may be conveyed to the place where a Vaccine patient resides, and the fluid may be transfered immediately from arm to arm; or a person who has the Vaccine affection may be conveyed to any place where the practice is to be introduced; and a considerable number may daily be inoculated from that single source.

But when it is impracticable to obtain fluid matter, there are various modes of transmitting it in a dry state. When it is to be used within two or three days, a lancet is a proper vehicle; but if it is not used within some such period, the lancet rusts, the matter becomes decomposed, and a spurious pustule is likely to be the consequence. This has been one of the most common causes of the numerous errors committed in Vaccine Inoculation. The Vaccine fluid is much more aqueous than the Variolous; and cannot, like the latter, be preserved on a lancet for any length of time without injury.

Many safe and effectual methods of preserving Cow-pock matter have been devised, and extensively practised. Some of the most common are, the taking of it on thread or glass, and suffering it to dry; then the thread or glass is to be charged again. This may be repeated several times. These methods are entitled to some degree of approbation; but they are attended with a great consumption of matter, which is important in an affection where that article is so sparingly produced.

Another objection to glass is, that many practitioners fail to infect their patients with virus thus preserved; either ther in consequence of diluting it too much, or not allowing it sufficient time to dissolve.

To remedy these inconveniences, other modes have been invented. Lancets have been made of gold, silver, platina, and ivory; which, when armed, have been employed for the transportation of Vaccine virus with advantage.

Another mode, which seems well calculated for general use, is to take the fluid on a quill, shaped like a tooth-pick, and suffer it to dry. At the time of inoculation, a puncture is first to be made with a lancet; then the point of the quill is to be inserted, and held in the part half a minute or more. Afterwards, the flat surfaces of the instrument may be repeatedly wiped on the place, in order to lodge as much matter as possible in the puncture.

To render this instrument the more portable, it may be made out of a slender portion of a quill, pointed at the end. This may be conveyed in a letter, or several of them may be enclosed within the barrel of another quill, stopped with white wax; sealing wax is unfit for the purpose; the heat necessary for melting it may decompose the matter.

Another method is, to take the matter on a small bit of ivory, shaped like the tooth of a comb, and pointed at the end like a lancet. This may be called a Vaccinator. It is well adapted to the purpose, since it easily penetrates the puncture made by a lancet; and is very portable, either in a common quill, or a crow quill, or wrapped in paper.

Would every practitioner take matter on these instruments, when he has a patient under Vaccine Inoculation, it would furnish him with a constant resource in case of necessity, both for himself and others.

Vaccine virus ought not to be taken after the areola begins to be extensive, nor to be dried before the fire, nor in any other way to be exposed to much heat.

We have enclosed some Vaccinators charged with matter, as a specimen of the method before recommended, and as a present supply.

We are preparing Medical Instructions for the new Society, which, we flattered ourselves, we should have been able to transmit; but the meetings of the Society have been so frequent, that we have had little time to devote to this purpose. When they are completed, they will be added to the laws of the Society, and shall be transmitted to your Excellency without delay.

We have the honour to be, &c.

EDWARD JENNER,
*London, March* 21, 1803. JOHN RING.

### A TABLE shewing the Advantages of VACCINE INOCULATION. By JOHN RING.

| THE NATURAL SMALL-POX. | THE INOCULATED SMALL-POX. | THE INOCULATED COW-POCK. |
| --- | --- | --- |
| I. The natural Small-pox is a loathsome, infectious, painful, and fatal disease. It is confined to no climate; but rages in every quarter of the world, and destroys a tenth part of mankind. | I. The inoculated Small-pox also is loathsome, infectious, painful, and sometimes fatal; and, when partially adopted, spreads the contagion, and increases the mortality of the disease. | I. The inoculated Cow-pock scarcely deserves the name of a disease. It is not infectious; and, in the opinion of the most experienced practitioners, has never proved fatal. |
| II. Those who survive the ravages of that dreadful distemper, often survive only to be the victims of other maladies; or to drag out a miserable existence worse than death. | II. It sometimes occasions the same maladies as the natural Small-pox. | II. It occasions no other disease. On the contrary, it has often been known to improve health; and to remedy those diseases under which the patient before laboured. |
| III. This cruel and lamentable disorder leaves behind it pits, scars, and other blemishes; and bodily deformities which embitter life. | III. It frequently leaves behind it the same blemishes and deformities as the natural Small-pox; which are the more deplorable as they were brought on by a voluntary act. | III. It leaves behind no blemish, but a *blessing*; — one of the greatest ever bestowed on man, — *a perfect security against the future infection of the Small-pox.* |

From this faithful statement of the advantages attending Vaccine Inoculation, it must appear evident to every unprejudiced person, that it is the duty as well as the interest of every parent, of every individual, and of every nation, to adopt the practice; and to hasten the Extermination of the Small-pox.

